# Comparing the cost‐effectiveness of drones, camera trapping and passive acoustic recorders in detecting changes in koala occupancy

**DOI:** 10.1002/ece3.11659

**Published:** 2024-07-02

**Authors:** Chad T. Beranek, Darren Southwell, Tim S. Jessop, Benjamin Hope, Veronica Fernandes Gama, Nicole Gallahar, Elliot Webb, Brad Law, Allen McIlwee, Jared Wood, Adam Roff, Graeme Gillespie

**Affiliations:** ^1^ Conservation Science Research Group University of Newcastle Callaghan New South Wales Australia; ^2^ Koala Science Team, Conservation and Restoration Science, Science, Economics and Insights Division New South Wales Department of Planning and Environment Parramatta New South Wales Australia; ^3^ Department of Primary Industries Forest Science Centre Parramatta New South Wales Australia; ^4^ NSW Wildlife Drone Hub, Vegetation and Biodiversity Mapping, Science, Economics, and Insights Division New South Wales Department of Climate Change and Energy Parramatta New South Wales Australia

**Keywords:** camera traps, detection probability, drones, passive acoustic recorders, power analysis, survey design

## Abstract

Quantifying the cost‐effectiveness of alternative sampling methods is crucial for efficient biodiversity monitoring and detection of population trends. In this study, we compared the cost‐effectiveness of three novel sampling methods for detecting changes in koala (*Phascolarctos cinereus*) occupancy: thermal drones, passive acoustic recorders and camera trapping. Specifically, we fitted single‐season occupancy‐detection models to data recorded from 46 sites in eight bioregions of New South Wales, Australia, between 2018 and 2022. We explored the effect of weather variables on daily detection probability for each method and, using these estimates, calculated the statistical power to detect 30%, 50% and 80% declines in koala occupancy. We calculated power for different combinations of sites (1–200) and repeat surveys (2–40) and developed a cost model that found the cheapest survey design that achieved 80% power to detect change. On average, detectability of koalas was highest with one 24‐h period of acoustic surveys (0.32, 95% CI's: 0.26, 0.39) compared to a 25‐ha flight of drone surveys (0.28, 95% 0.15, 0.48) or a 24‐h period of camera trapping consisting of six cameras (0.019, 95% CI's: 0.014, 0.025). We found a negative quadratic relationship between detection probability and air temperature for all three methods. Our power and cost analysis suggested that 148 sites surveyed with acoustic recorders deployed for 14 days would be the cheapest method to sufficiently detect a 30% decline in occupancy with 80% power. We recommend passive acoustic recorders as the most efficient sampling method for monitoring koala occupancy compared to cameras or drones. Further comparative studies are needed to compare the relative effectiveness of these methods and others when the monitoring objective is to detect change in koala abundance over time.

## Graphical abstract

This study evaluates the cost‐effectiveness of three sampling methods (thermal drones, passive acoustic recorders and camera trapping) for detecting changes in koala occupancy. Analysing data from 46 sites over 2018–2022, the study found that passive acoustic recorders deployed for 14 days at 148 sites provided the most cost‐efficient method (80% power to detect a 30% decline in occupancy). The study recommends passive acoustic recorders as the preferred sampling method for monitoring koala occupancy compared to drones or cameras.

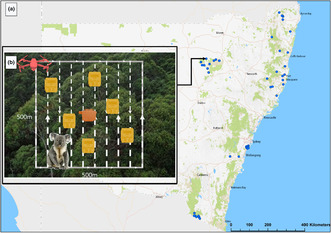

## 1 INTRODUCTION

Cost‐effective monitoring is crucial for determining the status and trends of plant and animal populations, as well as their response to threats or management intervention. Despite the importance of monitoring in ecology and conservation biology, most biodiversity monitoring programmes suffer from limited resources (Lindenmayer et al., 2012), which impose constraints on their size (number of sites), scope (single or multiple species), spatial scale, sampling intensity and the number and type of sampling methods. These constraints increase the need to design biodiversity monitoring programmes that maximise the probability of detection (i.e., minimise false absences) at sites while minimising cost and ensuring there is sufficient statistical power to accurately detect changes in the abundance or occupancy of target species over space and time (Kellner & Swihart, 2014; MacKenzie & Royle, 2005).

A key decision in the design of biodiversity monitoring programmes is the choice of sampling method. When choosing a method during the survey design stage, several factors should be considered, including cost, required experience or expertise, false‐positive/negative rates, data processing and storage, and the probability of detecting the target species (Caughlan & Oakley, 2001). Detection probability holds particular importance as it influences the statistical power to identify changes (e.g., occupancy or abundance) in populations over time or space (Guillera‐Arroita & Lahoz‐Monfort, 2012). Sampling methods with low detectability require greater monitoring effort to ensure adequate statistical power, either by increasing the number of sites or by spending more time surveying at each site (Field et al., 2005). Monitoring may have insufficient power to detect any changes of interest in a population over time when detectability is low. Failing to detect changes in populations is especially problematic if a species is at imminent risk of extinction or if it leads to false conclusions or delayed decisions about the effectiveness of management actions.

Recent advances in technology are providing opportunities for more cost‐efficient data collection and analysis in biodiversity monitoring (Stephenson, 2020). For example, technologies such as drones, camera traps and passive acoustic devices (PARS) have enabled researchers and practitioners to gather vast amounts of data with greater efficiency at lower costs (Lahoz‐Monfort & Magrath, 2021). Such technologies allow for monitoring large and inaccessible areas and, in the case of passive devices such as camera traps and PARs, provide high‐resolution sampling across large spatial and temporal scales (Han et al., 2015; Hill et al., 2018). Advances in complementary analytical pipelines, such as machine learning, have led to further efficiencies in data processing from electronic‐based ecological monitoring platforms (Tuia et al., 2022). Despite the promise of these technological advances, it is important to consider the precise objectives of a monitoring programme and the relative cost‐effectiveness of alternative sampling approaches in the survey design before implementation.

The koala (*Phascolarctos cinereus*) is an iconic arboreal marsupial found in open forest and woodland communities in south‐eastern Australia (Shumway et al., 2015). The species is currently listed as endangered, with many populations in decline due to a range of threats such as habitat loss and fragmentation, introduced predators, collisions with vehicles and disease (McAlpine et al., 2015). Management of koalas can be complex – in some regions, populations have become overabundant, leading to population collapses when food resources are depleted (Martin, 1985) and broader ecosystem‐wide impacts (Whisson & Ashman, 2020), while in other areas populations require conservation management and habitat preservation to prevent local extinction (Law, Gonsalves, Burgar, Brassil, Kerr & O'Loughlin, 2022; Lunney et al., 2014). Given a conservation status of endangered across much of the geographic range and uncertainty surrounding their response to management, cost‐effective monitoring is needed to determine how koala populations are trending over time so that koala conservation and ecosystem health can be maintained.

There are many traditional approaches and emerging technologies for surveying koala occupancy, activity, and abundance (Howell et al., 2021). Early koala surveys consisted of diurnal transect surveys (Martin, 1985), nocturnal transect surveys with a spotlight (Catling et al., 1997), or scat surveys (Lunney et al., 1998; Munks et al., 1996). More recent studies have explored the use of scat searches being implemented with detection dogs (Cristescu et al., 2015), PARs (Law et al., 2018) and drone surveys using a thermal imagery camera payload (Beranek et al., 2020). However, there have been few attempts to compare the relative effectiveness of these methods. For example, a recent study indicated that nocturnal transects using spotlights have a higher detection rate compared to diurnal transects (Wilmott et al., 2018). While another study found the detection probability from PAR surveys was much higher than spotlighting surveys (Goldingay et al., 2022). Drone surveys are more effective at counting koalas in quadrat‐based surveys compared to spotlight transects and grid‐based scat surveys; however, differences in koala occupancy obtained between each method were unclear, with inadequate sample sizes to draw firm conclusions (Witt et al., 2020). Scat surveys conducted with detection dogs were more efficient than those conducted with human observers (Cristescu et al., 2015). Camera traps have not been evaluated as a survey tool for koalas despite their widespread use for surveying a suite of other vertebrate species.

To our knowledge, no study has yet assessed the relative efficacy of contemporary and emerging survey techniques to detect changes in koala occupancy, such as drones, camera traps and PARs, using data collected at the same survey sites. Therefore, the aims of this study were threefold. First, we estimated and compared the detection probability of three emerging sampling approaches for koalas (camera trapping, PARs, and thermal drone surveys) using detection/non‐detection data collected from three concurrent surveys in New South Wales, Australia. In doing so, we quantified how environmental and weather conditions affected koala detection probability for each method. Second, using our estimates of detectability combined with mean estimates of site occupancy, we estimated the statistical power to detect a change in koala occupancy (at a rate of 30%, 50% and 80% decline) for each sampling method across a range of survey designs (i.e., combinations of the number of sites and the number of days each method was deployed). Thirdly, we developed a cost model to determine the cheapest method and survey design needed to have at least 80% statistical power to detect each decline in occupancy. We provide discussion regarding how these results may be used to improve effectiveness and efficiencies in the design of koala survey and monitoring strategies at large spatial and temporal scales.

## 2 MATERIALS AND METHODS

### 2.1 Study area and sampling design

**FIGURE 1:**
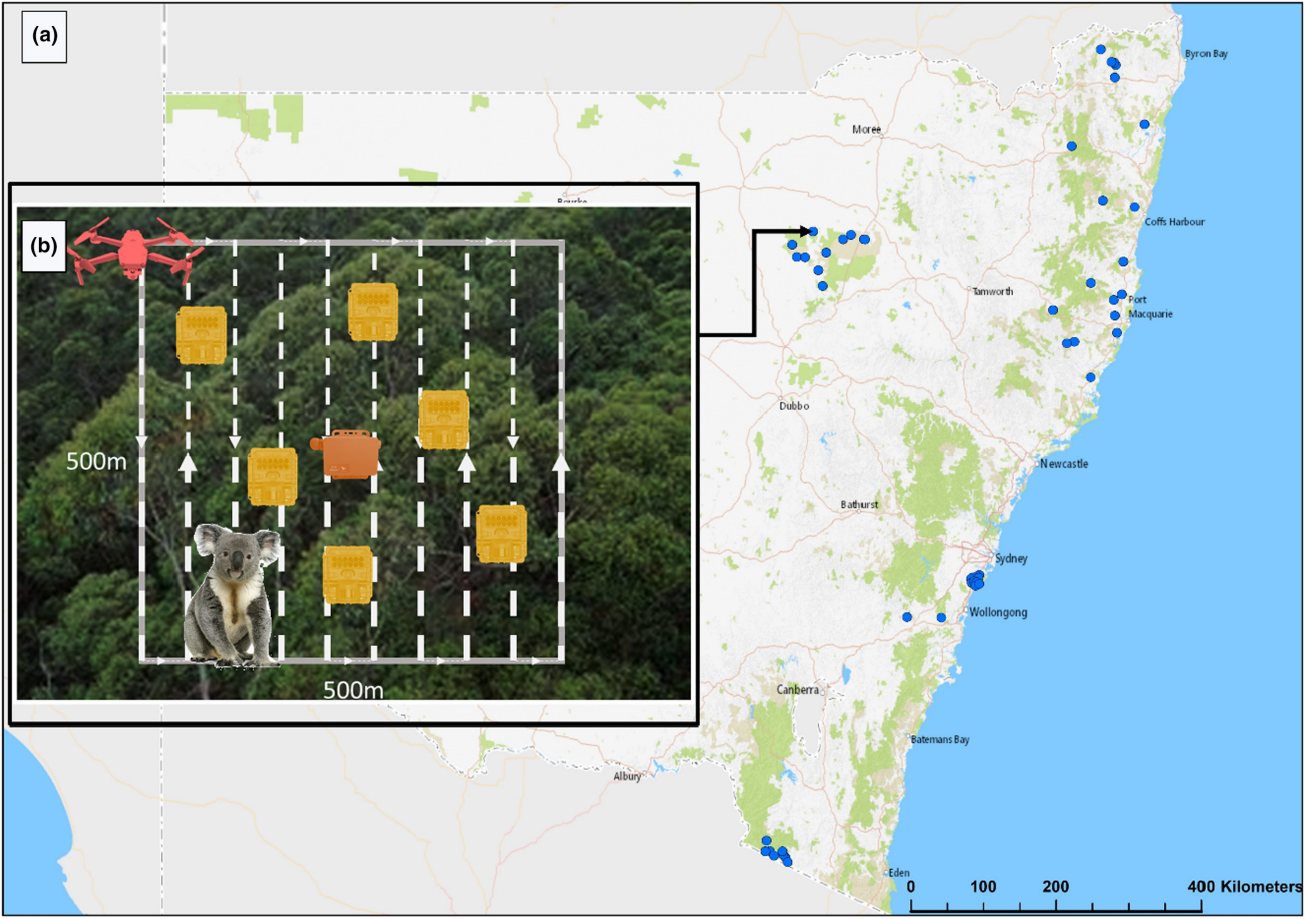
Location (a) of koala survey sites distributed across New South Wales. The inset (b) depicts a schematic of the site area and the method‐specific survey sampling design and deployment for drones (red), six camera traps (yellow), and an acoustic song meter at the site centroid (orange).

Our study was conducted in tall wet sclerophyll forests, dry sclerophyll forests and woodlands of New South Wales, Australia (Figure 1). In total, 46 sites were surveyed covering eight bioregions and a range of habitat types and topographic features thought to drive koala distributions. All sites were in National Park and State Forest, except for one on private land in the Southern Highlands. Thirty‐eight of the 46 sites were aligned with recent PARs surveys that either detected koalas or were assessed to be in high‐quality habitat (Table S1). The remaining nine sites were randomly selected within Royal National and Heathcote Parks and had not been surveyed recently. All sites were no closer than 5 km apart and were close to roads to maximise accessibility. Each site contained a 25‐ha plot (500 m × 500 m), selected to encompass the potential home ranges of multiple koalas and to be consistent with previous drone‐based study designs.

### 2.2 Koala survey methods

At each site, three survey methods (Figure 1b) were used to detect koalas:

#### 2.2.1 Drone surveys

A quadcopter drone (DJI Mavic2 Enterprise Advanced) mounted with 12MP wide camera, 48MP zoom camera, 640 × 512px 30fps thermal camera was used to survey koalas from March to August 2022. A mean of 2 ± 0.42 SD repeat drone surveys was conducted per site. All drone surveys were flown at a fixed speed of 8 m/s and at a height no greater than 60 m above the ground (approximately 30 m above the top of the canopy). Drone surveys conducted a preprogrammed lawn mower flight pattern (parallel linear line‐transects) over each site with 10% overlap to provide total site survey coverage. Pilots were able to interrupt a flight to manually investigate potential koala sightings. All surveys were conducted at night (8 pm–3 am) to coincide with temperatures below 18.0°C (range 6.0–18.0°C) to increase the contrast between the body temperature of koalas and their surroundings to improve detection. Surveys were only conducted when wind speeds were below 15 m/s, and there was an absence of rain, cloud or fog that could limit visibility. Each plot was repeated on the same night by a different pilot.

Drone‐derived survey footage was viewed in real time by a single observer by watching footage on a 14 cm widescreen DJI RC plus remote controller. All arboreal species were first detected from their thermal signature and behaviour using the drone's thermal camera and the detection locality was recorded as a waypoint. Two methods were used to verify possible koala thermal detections: either (1) the drone was flown in manual mode to gain closer inspection of the animal to determine the species, or (2) ground‐based observers using spotlights, binoculars and handheld FLIR E86 (24° lens) cameras inspected each potential koala detection. All detections and survey‐relevant covariates were recorded in real time using a customised data entry proforma built‐in Survey123.

#### 2.2.2 Passive acoustic recorder surveys

Most acoustic survey data (38 of 46 sites) were compiled from recently conducted surveys of koalas conducted between 2019 and 2022 using comparable sampling methodology. These surveys were conducted by the Department of Planning and Environment (Science Economics and Insights and National Parks and Wildlife Service), DPI Forest Science, NSW Forestry Corporation, Southern Cross University and Australian National University.

A single acoustic sensor (using one of the following passive acoustic recorder models: Song Meters SM4, SM3, or Mini/ Wildlife Acoustics) was deployed at the centroid of each site to acoustically detect the presence of koalas. Song Meters were fixed to trees using cable ties at a standard height of 150 cm. Song Meter deployment ranged between 8 and 42 days (mean: 17 days ± 12 SD) during spring and early summer to sample during the breeding season when male koalas are most vocal (Law et al., 2018, 2020). Song Meters were programmed to record from sunset until sunrise (North Coast, Pilliga State Forests), the peak calling period of koalas (Ellis et al., 2011; Hagens et al., 2018; Law et al., 2020), using a sampling rate of 22,050 Hz, and a resolution of 16 bits per sample. Song Meters in Richmond Range NP were set to record for 5 h after dark (Goldingay et al., 2022). Song Meters are considered to have up to a 300 m omnidirectional sampling radius in ideal conditions, allowing them to potentially sample 100% of the site area (Law et al., 2018). However, playback field experiments performed by Hagens et al. (2018) report a loss of quality at 100 m, which can be further reduced in denser habitat.

Koala call activity was obtained via two methods. The first method consisted of manually recording koala bellows from spectrograms, which was only used in Richmond Range sites. The second method used koala recognisers developed by the NSW Department of Primary Industries (Law et al., 2018; Koala_CNN_LG_010822 and DPI_Male_Koala_V3_CNN15_10–02‐23), run in the AviaNZ software. The recognisers were built primarily to detect male calls, but they sometimes detect females. Males and females were included with no distinction in call activity data and presence/absence data.

#### 2.2.3 Camera trap surveys

Six cameras per site were deployed, spaced at 100–150 m intervals along two parallel transects. We used Reconyx HP2W Hyperfire 2 Professional White Flash (Professional Trapping Supplies) cameras powered by Eneloop NiMH, with 32 GB memory cards. Deployment was between 2 May and 28 July 2022 for between 67 and 264 days (mean: 99 days ± 39 SD). The following settings were used: three photos per trigger, rapidfire, no latency period, sensitivity was set to high, 4:3 resolution, high frequency PIR, day/night, 1/480 shutter and ISO 3200. Variability in camera deployment time was due to camera malfunction and logistic constraints in camera collection at some sites.

Camera locations were assigned based on a desktop review and were placed across contours in the dominant habitat type present in the survey grid. Each camera was secured to a tree 45 cm above the ground and the centre of the photo aimed at a point on the ground 3 m from the tree trunk. The aim was precisely adjusted by reviewing images with a handheld camera with the centre accurately marked to ensure a consistent field of detection with survey teams instructed to follow the specifications to ensure consistency. All ground and low‐shrub vegetation between the camera and the focal point, and at least 1.5 m behind the focal point, was cleared. No lure was used.

All photographic images were reviewed independently by at least two ecologists familiar with Koalas, and these images were extracted for analyses. Where observer's tags disagreed, the images were reviewed for final species determination. The images were tagged with EXIF tags (EXIFpro 2.0) to identify the following taxa: koala, wombat, fox, dog, cat, other mammal, bird, other animal, reptile and no animal. The start and end images and any camera disturbance events were also tagged and checked against other information to ensure times and dates were correct. Where cameras reported a high number of false triggers (typically greater than 300 images per day) or did not record images beyond two‐week deployment durations, their data were not included for analysis. Exif information was then extracted (R version 4.1.0 using the package exiftoolr), and a daily detection/non‐detection history of koalas was made for each site.

### 2.3 Observational covariates

We extracted data on the relative humidity (%), maximum air temperature (°C) and rain on the day (mm) of each survey. These covariates were extracted from SILO (https://www.longpaddock.qld.gov.au/silo/) which spatially interpolates weather data across Australia at a 5 × 5 km resolution. All data were transformed into *Z*‐scores to enable direct comparison of model coefficients.

### 2.4 Data processing

Koala detections from each sampling method were collapsed into 24‐h bins. A detection for camera traps was considered as having at least one validated photo of a koala from any of the six camera traps during a 24‐h period (from 12 AM). A detection for acoustic surveys was considered as having at least one validated koala bellow from one PAR during 24 h (from 12 AM). A detection for drone surveys was considered as observing at least one koala thermal signature during a complete flight across a quadrat.

### 2.5 Single‐visit detection probabilities

We modelled detectability of koalas with a single season site occupancy‐detection model (MacKenzie et al., 2002), which assumed the result from any given survey was the outcome of two binomial processes acting simultaneously: (1) the probability a koala was present at a site (*ψ*) over long time periods; and (2) the probability a koala was present within the site and observed in any given survey visit (*ρ*). Given our focus was on estimating detectability, we analysed all three methods together in the same model, represented by a categorical variable “method” and held the occupancy parameter ψ constant. Under this modelling framework, we assumed that there was no change in occupancy status between surveys and between the sampling periods of each method. We also evaluated the linear and quadratic effects of relative humidity and maximum air temperature and a linear effect of rainfall on koala detectability. Since the response of each detection covariate was likely to differ between sampling methods, we only considered interactions between the sampling method and weather variables. We considered it plausible that any of the weather covariates could be predictive of koala detection probability either singly or in a multi‐variate model. Hence, all covariates were assessed in single models and then with all possible combinations of models containing two covariates, resulting in a set of 15 candidate models (see Table S2 in the supplementary materials for the model descriptions).

We fitted occupancy‐detectability models using the *unmarked* package in R (Fiske & Chandler, 2011) and used Akaike's information criterion (AICc) to assess the fit of each model; the model with the lowest AICc value was considered most parsimonious. Model fit was evaluated using two different tests with 10,000 bootstraps: sum of squared errors and Freeman‐Tukey chi‐square (see Table S3 and Figure S1). We used an alpha value of 0.05 to determine whether there was a lack of fit. This was performed for the null model and all models with a ΔAICc <2.

### 2.6 Cumulative detection probabilities

We calculated the cumulative detection probability $P^{*}$ for each method using the top model from above, given by:

(1) $$P^{*}=1-{\left(1-p\right)}^{K}$$

where *p* is the mean detection probability for a day/night of surveying and *K* is the number of repeated days/nights of surveying. The detection probability curve depicts the number of repeat surveys (in this case 24‐h periods) required to result in a given probability of detecting the species at a site where it is present (Kéry, 2002).

### 2.7 Power analysis

We used the power analysis equation outlined in Guillera‐Arroita and Lahoz‐Monfort (2012) to estimate the statistical power of each sampling method to detect a change in koala occupancy, given decisions about the magnitude of a change in occupancy (*R*), the number of sites surveyed (*S*) and the number of sampling occasions (*K*). In this equation, statistical power *G* is given by:

(2) $$G=1–\beta =\left\{1-\phi \left(\frac{Z_{\alpha /2}\sqrt{\sigma_{1}^{2}+\sigma_{2}^{2}}-\left(\psi_{1}+\psi_{2}\right)}{\sqrt{\sigma_{1}^{2}+\sigma_{2}^{2}}}\right)\right\}+\phi \left(\frac{Z_{\alpha /2}\sqrt{\sigma_{1}^{2}+\sigma_{2}^{2}}-\left(\psi_{1}+\psi_{2}\right)}{\sqrt{\sigma_{1}^{2}+\sigma_{2}^{2}}}\right)$$

where *ψ*_*1*_ and *ψ*_2_ are equal to the occupancy probabilities before and after a change (*R*), *Z*_α/2_ is the upper 100α/2‐percentage point for the standard normal distribution, $\phi \;\left(x\right)$ is the cumulative distribution function for the standard normal distribution, and $\sigma_{i}^{2}$ = (1 – *O*_*i*_ + *F*_*i*_)/*S*_*i*_ where:

(3) $$F_{i}=\left(1-p^{*}\right)/{\left\{p^{*}-\mathrm{Kp}\left(1-p\right)\right\}}^{K-1}$$

Here, *p* is the single‐visit detection probability (1 day/night), *K* is the number of repeat visits and *S* is the number of sites. We set alpha α to 0.2 as it better reflects the costs of committing a Type II error (failing to detect a decline), which, for threatened species, could lead to extinctions (di Stephano, 2003).

### 2.8 Alternative survey designs

Using Equations 2 and 3, we calculated the statistical power (*G*) for each sampling method for different combinations of repeat surveys (*K*), number of sites (S) and proportional changes in occupancy (*R*) given our single visit estimates of detection probability for drones (one flight), camera trapping and acoustic surveys (24 h period). We estimated power for all combinations of sites (*S* = 1–200), repeat surveys (*K* = 2–20) and changes in occupancy (*R* = 30%, 50% and 80% declines), giving a total of 11,400 survey design simulations for sampling method. Our magnitudes of change were chosen to reflect decline thresholds as defined by IUCN criterion A2c for classifying a species as threatened; a reduction in occupancy of ≥80% (Critically Endangered), ≥50% (Endangered), or ≥30% (Vulnerable) over whichever is longer out of any 10 years or three generations (as per Moore et al., 2023). The initial occupancy was obtained from the top‐ranked occupancy detection model.

### 2.9 Survey cost

We developed a cost model to estimate the total cost of implementing the alternative survey designs described above. Total cost *C* for each survey method was calculated as:

(4) $$C=\left(\mathrm{UC}\right)+\left(S\times \mathrm{CS}\right)+\left(S\times K\times \mathrm{CSS}\right)$$

where UC is the startup costs and includes equipment, training and licencing, *S* is the number of sites, and CS is the cost per site and includes equipment, planning, maintenance, personnel and travel. *K* is the number of repeat surveys and CSS is the cost per site and survey and includes post‐processing of data (for cameras and PARs) and travel and personnel costs for drones. The cost breakdown for each method is provided in Table S4. We did not include the costs it took to develop recognisers for koala bellows into the startup costs for PARs since this was already available at the inception of this study.

## 3 RESULTS

### 3.1 Koala detections

A total of 143 koala detections were obtained using all three survey methods. Most koala detections were reported from PARS, with 75 acoustic detections obtained from 619 surveys (each survey consisting of 24 h bins). Camera traps had 47 detections from 4563 surveys (each survey consisting of 24 h bins of the six camera traps deployed at a site). Drone surveys totalled 21 detections from 89 surveys. Examining koala detections at sites on regional basin, we found 3/10 sites with koala presence in Pilliga State Forests (only detected via acoustics), 2/9 sites in Heathcote/Royal National Parks (this site was not surveyed by acoustics; *n* sites with koala detections via camera = 1, and via drone = 2), 10/13 sites in north‐eastern forests (*n* sites with koala detections via acoustics = 6, via camera = 9, via drone = 5), 5/8 sites in Kosciusko NP (*n* sites with koala detections via acoustics = 5 acoustic, via camera = 2 and via drone = 1), 1/2 sites southern highlands (*n* sites with koala detections via acoustics, camera and drones = 1), and lastly, 3/4 sites in Richmond Range (*n* sites with koala detections via acoustic, camera and drones = 3). The detection probability per 24‐h bin for each method per survey event was 0.31 for acoustic surveys (95% CI's: 0.24, 0.38), 0.017 for camera trap surveys (95% CI's: 0.013, 0.023) and 0.28 for drone surveys (95% CI's: 0.13, 0.62). The probability of koala occupancy was estimated to be 0.54 (95% CI's: 0.52, 0.67).

### 3.2 Occupancy‐detection model

**FIGURE 2:**
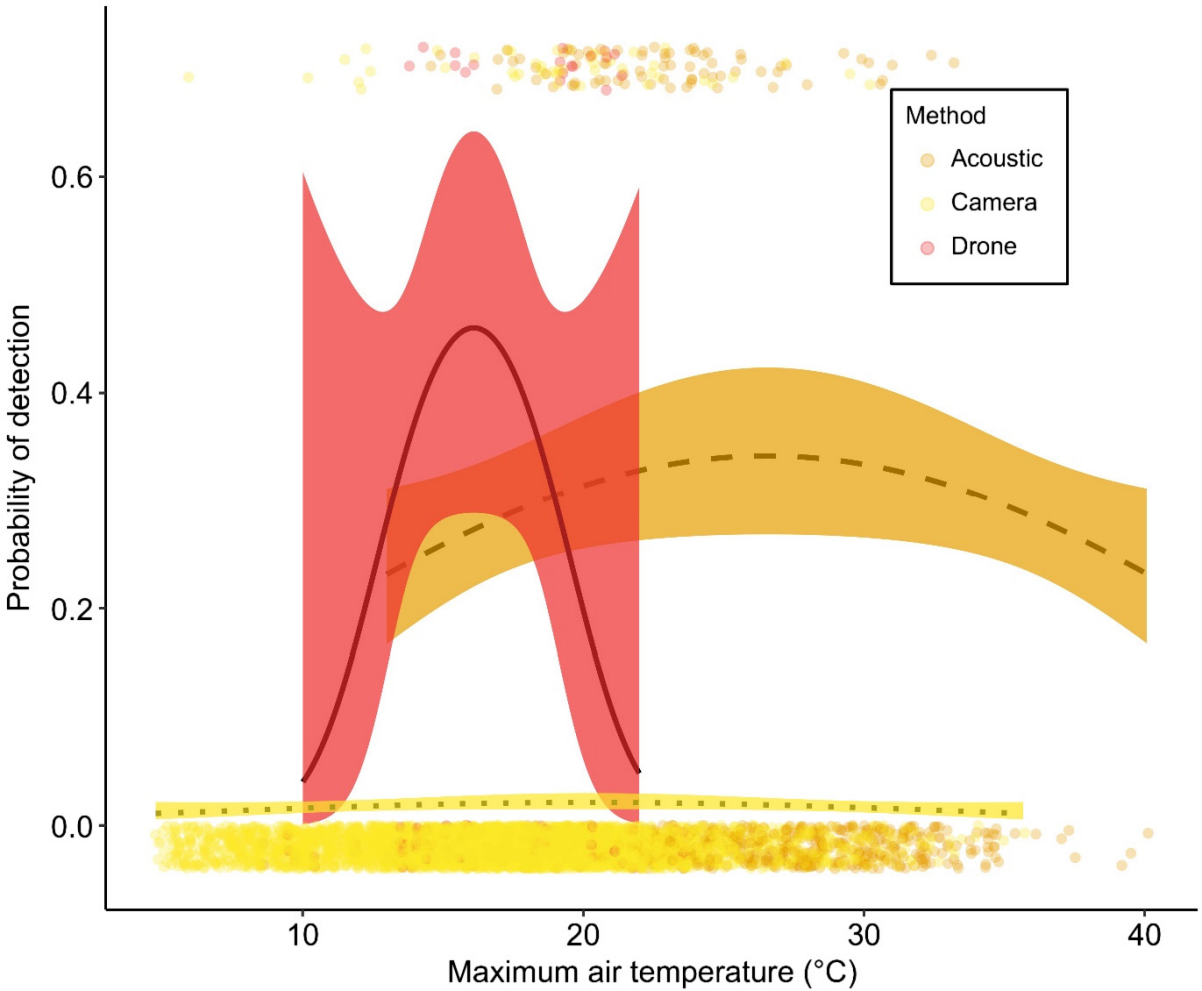
The influence of maximum daily air temperature on method‐specific koala detection probability. Predictions are based on the most parsimonious model. Error bars indicate 95% confidence intervals. Points above curves indicate koala detections and points below the curve indicate where a survey did not detect a koala. The prediction curves are bounded to only include the minimum and maximum extents of where data were obtained for each method.

There was one model that provided substantial support (ΔAICc ≤2) to best explain variation in koala detection probability. The most parsimonious model provided evidence of an interactive effect between method and a quadratic influence of air temperature on koala detection probability. This model predicted a concave‐up relationship between temperature and koala detection probability for each survey method (Table 1 and Figure 2).

**TABLE 1:** Ranking of the top 10 models evaluating the effects of different covariates on koala detection probability estimated using single‐season occupancy models.

Model	*n*	AICc	ΔAICc	Cumulative weight
M7: *p*(_method × temp2_) ψ_(.)_	7	926.06	0	0.61
M6: *p*(_method × temp_) ψ_(.)_	7	928.47	2.41	0.79
M4: *p*(_method × rh_) ψ_(.)_	7	930.25	4.19	0.87
M2: *p*(_method_) ψ_(.)_	4	930.91	4.85	0.92
M11: *p*(_method × rain + method × temp2_) ψ_(.)_	10	932.28	6.22	0.95
M12: *p*(_method × rh + method × temp_) ψ_(.)_	10	933.68	7.62	0.96
M15: *p*(_method × rh2 + method × temp2_) ψ_(.)_	10	934.28	8.22	0.97
M13: *p*(_method × rh + method × temp2_) ψ_(.)_	10	934.62	8.56	0.98
M14: *p*(_method × rh2 + method × temp_) ψ_(.)_	10	935.38	9.32	0.99
M10: *p*(_method × rain + method × temp_) ψ_(.)_	10	935.68	9.62	0.99

*Note*: Column headings include *n*, the number of estimated parameters. AICc, Akaike Information Criterion; ΔAICc, difference between the AICc of the target model and the most parsimonious model. See Table S2 for model descriptions.

### 3.3 Cumulative detection curves

**FIGURE 3:**
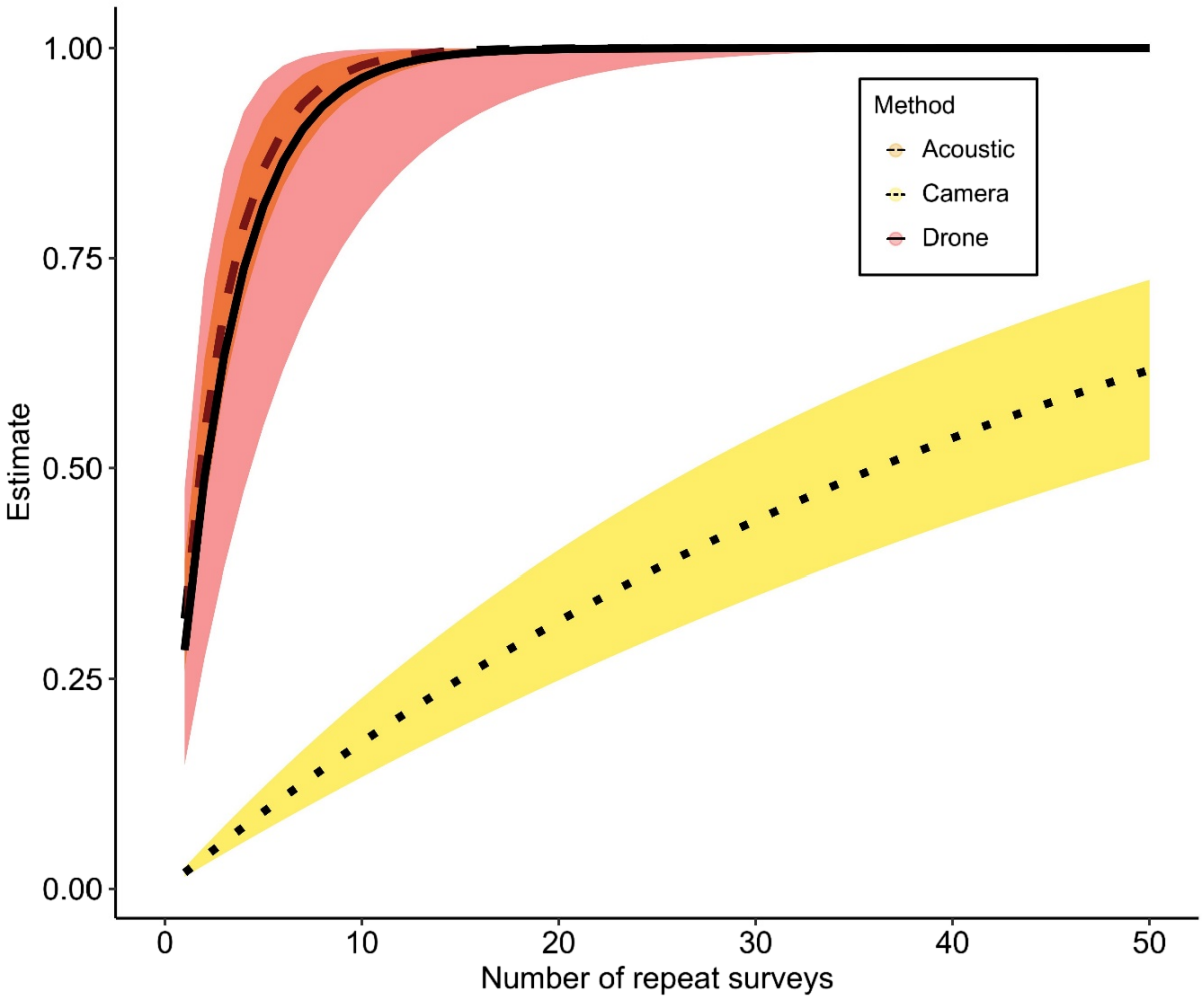
Koala cumulative detection probability curves for each method. Each survey for drone surveys comprised of the entire 25 ha plot. Each repeat survey for PARS and camera traps is one 24 h period of field deployment.

Passive acoustic devices had the smallest number of repeat surveys required to achieve a 95% confidence of detecting koalas (*K* = 7.72 days of deployment, 95% CI's: 6.05, 9.96). This was followed by drones (*K* = 8.97 repeat surveys, 95% CI's: 4.64, 18.74) and camera trap surveys (*K* = 156.14 days of deployment, 95% CI's: 116.28, 209.83). See Figure 3 for a comparison of detection curves for each method.

### 3.4 Power to detect change in occupancy

**FIGURE 4:**
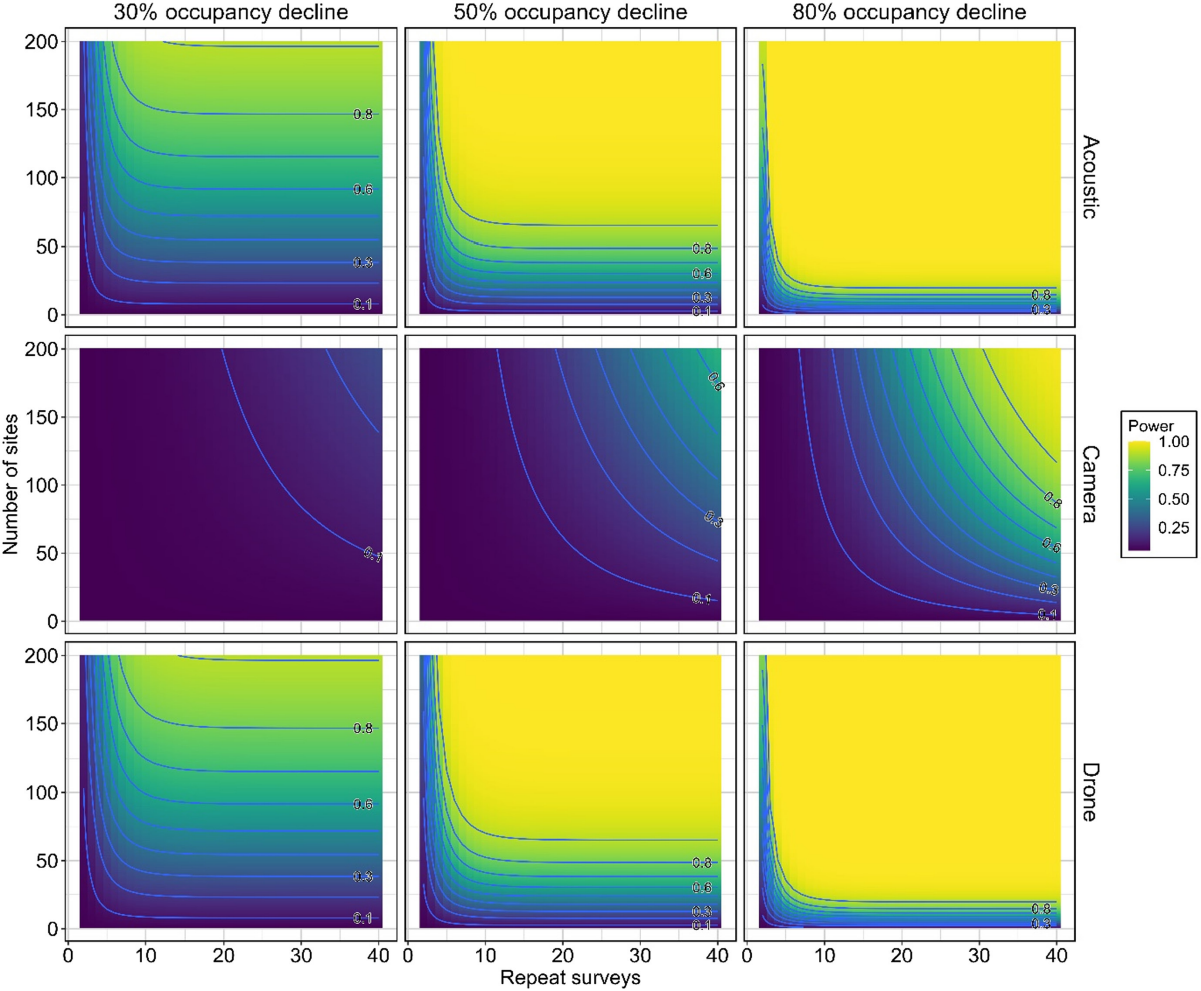
Heatmap plots depicting method‐specific relationships among survey effort, number of sites and associated power to detect declines in koala occupancy of 30%, 50% and 80%. Contour lines have intervals of 0.1 values of power. Each survey for drone surveys is one lap of the entire 25 ha plot. Each repeat survey for acoustic and camera traps is one 24‐h period of field deployment.

There was wide variation in the number of sites, and repeat surveys were required to have an 80% power to detect a decline between each method (Figure 4). PARS required the least number of repeat surveys to achieve maximum power to detect all scenarios of occupancy decline. With this method, 6 days at 200 sites would be needed to have 80% power to detect a 30% decline. The number of repeat surveys required by camera trap and drone surveys was variable, and the minimum number of repeat surveys required depended on which decline percentage was required to be detected for all methods. Camera traps required more than 40 days of deployment across 200 sites to detect a 30% or 50% decline with 80% power. For camera traps to detect an 80% occupancy decline with 80% power, a deployment duration between 31‐days (across 200 sites) to 40‐days (across 128 sites) was required. The minimum number of repeat surveys with drone surveys required to detect 30%, 50% and 80% decline in occupancy with an 80% power was seven repeat surveys (across 200 25‐ha quadrats), three repeat surveys (across 180 25‐ha quadrats), and two repeat surveys (across 130 25‐ha quadrats) respectively.

### 3.5 Survey cost

**FIGURE 5:**
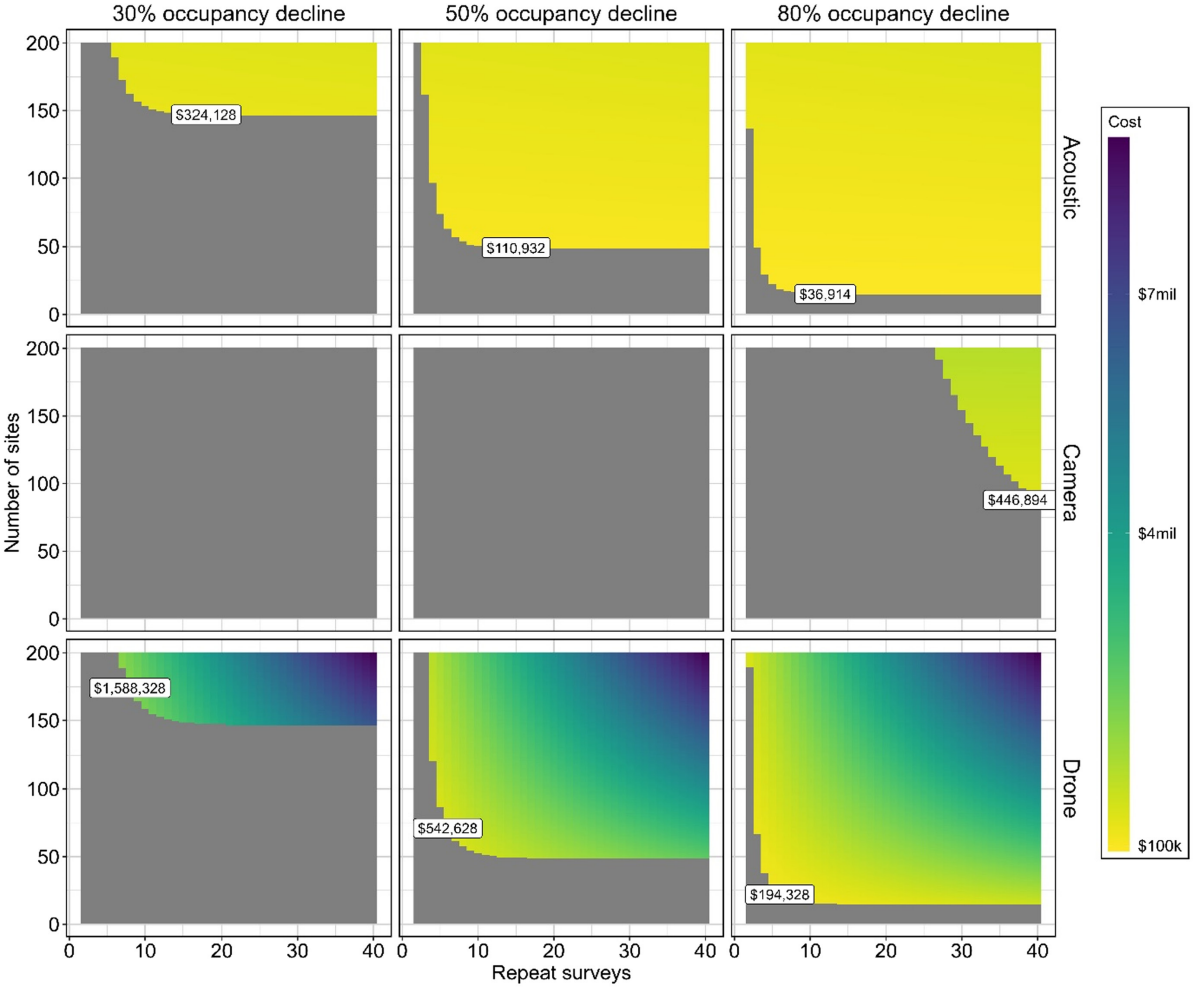
Heatmap plots depicting method‐specific costs among different survey effort and number of sites monitoring scenarios to detect declines in koala occupancy of 30%, 50% and 80%. Only costs within an 80% power threshold to detect decline are presented. Each survey for drone surveys is one lap of the entire 25 ha plot. Text boxes indicate the most cost‐effective number of sites and repeat survey combinations in each method and decline scenario. Each repeat survey for acoustic and camera traps is one 24‐h period of field deployment.

The cheapest method to detect a change in occupancy with at least 80% power across all decline scenarios considered was PARs (see Figure 5). Only drones and PARs had a high enough power to detect a change in occupancy of 30%, but PARs provided the cheapest sampling effort to achieve 80% power, being 5 times cheaper ($1,588,328 and $324,128, respectively). This trend continued in the scenario of detecting a 50% change in koala occupancy ($485,828 and $110,932, respectively). Camera traps were the most expensive method to detect an 80% change in koala occupancy ($446,894) which was 2.3 times more costly than drones ($194,328) and 12.1 times more costly than PARs ($36,914). The cheapest PAR survey design for detecting a 30% decline in occupancy included 148 sites with a deployment length of 14 days.

## 4 DISCUSSION

Quantifying the cost‐effectiveness of alternative survey methods is crucial during the design stage of a biodiversity survey or monitoring programme. This study demonstrates the capacity of different survey methods, and environmental covariates, to strongly influence koala detection probability. In turn, this resulted in method‐specific survey design considerations that would be needed to reliably detect declines with sufficient power with the least cost to monitor koala occupancy. We discuss these results regarding potential explanations for differences observed between methods, optimising experimental design, study limitations and finally suggest future directions to increase our understanding of optimising survey design for koalas.

### 4.1 Effect of sampling method on koala detection probability

A key result from the occupancy modelling was that the survey method strongly influenced koala detection probability. PARs and drones provided the highest koala detection probability compared to cameras. The spatial–temporal variation in abundance of the target species and how this interacts with the spatial–temporal variation in the application of a given survey method is likely to explain these results. For example, key differences in these factors intuitively explain why PARs and drones achieved a higher koala detection probability compared to cameras. PARs have a high detection probability since a single male koala can bellow frequently nightly and over several weeks, and PARs are usually deployed for several weeks, satisfying the temporal component of increasing detection. In addition, male koalas frequently move around, especially in breeding season REF, which increases the chance that koala bellows will be recorded by a PAR that is deployed for a long duration. Each bellow is audible from large distances (~300 m radius), satisfying the spatial component of increasing detection. Drones have a high detection probability since they can cover a wide spatial area (~25‐Ha) and are also able to detect female koalas, yet they require repeat surveys to increase their temporal coverage.

Our results show a lower detection rate compared to other studies that used PARs. The detection curve of Hagens et al. (2018) highlighted that deployments of 7–10 days were required to detect koalas in the early breeding season whereas 2–5 days were required in the late breeding season. Similarly, Law et al. (2018, 2020) found a detection probability per night of 0.35–0.45, which yields a detection probability >90% for seven nights of sampling. Goldingay et al. (2022) estimated a nightly detection probability of 0.79 which equates to only two nights to achieve 95% prob of detection. Our results revealed that longer deployments were required (6–11 days) to have 95% confidence in detecting koalas. This difference could be due to the differences in population densities, sex ratios or environmental or social‐related differences between the populations studied.

Acoustic‐based detection probability is expected to increase with koala population density, in male‐biased populations with stronger male–male competition or where the energetic costs of calling are reduced due to environmental productivity. The study by Hagens et al. (2018) was conducted on some populations that are considered highly abundant, such as in Cape Otway (Whisson et al., 2016: Cape Otway 10–18 per hectare). In comparison, most populations included in the present study had relatively low densities (Law et al., 2021: Canyonleigh 0.02/ha, Upper Nepean: 0.06/ha, Goldingay et al., 2022: 0.14–0.18/ha, Law, Gonsalves, Burgar, Brassil, Kerr & O'Loughlin, 2022: Kalanteenee 0.04–0.055/ha). Hence the density of the target population and how this relates to detection probability is an important consideration for ecological monitoring programmes, especially for species like the koala which persist at vastly different population densities across their distribution. The occupancy of the target population is also critical given detecting a 30% decline is easier to detect if occupancy starts at larger values such as 0.65–0.8 (Goldingay et al., 2022).

Our camera monitoring design (i.e., 6 camera traps/site) was based on other studies which have used a similar design known to be effective at detecting rare and cryptic mammals such as feral cats (Stokeld et al., 2015) and quolls (Heiniger et al., 2020). Nevertheless, even though cameras had the longest deployment durations, the cumulative spatial sample coverage (150 m^2^ total) and hence survey effort of the site camera array was comparatively small. Importantly, camera detection efficacy would be directly influenced by the interaction between koala abundance and the typically limited distance and duration of terrestrial activity (Ellis et al., 2011). These two attributes would explain the poor detection probability obtained by cameras.

It is conceivable that alternative camera deployment designs such as positioning a camera to focus its field of view on the trunks of the most important food tree species (i.e., key koala micro resources) within a site, or the use of pheromonal lures, could allow for improved camera detection performance (Mills et al., 2019). Furthermore, ensuring the cameras were deployed in the early breeding season in spring when koalas are more likely to move greater distances on the ground could allow for greater detection probability (Ellis et al., 2011; Ryan et al., 2013). However, these camera sampling alterations would need to lead to substantial improvement in koala detection probability to justify their use for koala occupancy monitoring compared to other methods. Nevertheless, use of camera monitoring might be considered valuable if it provided a tool to collect ancillary ecological information (e.g., presence of predators) to aid interpretation of patterns in koala occupancy derived by more effective survey methods (Goldingay et al., 2022).

Drones produced comparable per night koala detection probability to PARs, and with a similar detection curve, where about nine repeat surveys are needed to ensure high certainty of koala detection within a site. A major benefit is that data derived from drone surveys are instantly obtained, provide count data, identify exact real‐time locations of the target species and do not require post‐processing, and hence, drone surveys provide the best rapid survey method to determine koala presence within a site. Nevertheless, because of their associated costs, optimal survey effort for long‐term monitoring with this method can be cost‐prohibitive, where drones are 4.5 times greater than PARs. A limitation of this study, again reflecting cost and time constraints, is that drone surveys were not repeated enough to gain precise measures of detection probabilities (*n* = ~2 repeats at each site). This warrants some caution in interpreting the results of the power to detect change in occupancy for drone surveys. Further research is required with larger numbers of repeat drone surveys or larger survey plots to refine estimates of detection probability and power to detect change in occupancy when surveying for koalas with drones.

### 4.2 Effect of observational variables on koala detection probability

Beyond the effects of survey method, koala detection probability appeared to be influenced by air temperature. Indeed, all survey methods exhibited a concave‐down thermal dependence in koala detection with highest detection probabilities associated with intermediate daily maximum air temperatures. A negative relationship has previously been recorded for minimum temperature (Law et al., 2018), rainfall (Law et al., 2020), topographic position in some regions (Law et al., 2020) and a small decrease during the full moon (Law et al., 2018). These relationships may be useful to inform daily and seasonal timing of surveys to optimise detection if temperature influences koala behaviour (e.g., calling or movement) or if it increases method‐specific sensor performance that leads to higher koala detection. For example, trade‐offs between the costs of bellowing might favour increased bellowing at intermediate temperatures when thermoregulatory costs are reduced resulting in increases of acoustic‐based detectability. However, given the large geographic extent over which the study occurred, we cannot discount that this relationship may simply represent association between underlying koala population density and broadscale temperature and elevation differences.

### 4.3 Costs and power to detect change in occupancy

Our power and cost analyses informed how to optimise the survey design (i.e., number of sites versus number of repeats) for each method to detect change in occupancy between years. The cost of PARs stayed relatively low as the number of sites and/or repeats increased. This is because the recorder units are relatively cheap to purchase and increasing the deployment time of units incurs very little additional cost. However, given PARs have a relatively high detection probability, we recommend increasing the number of sites instead of increasing deployment time as there are no gains in power to detect change after deployment of 2 weeks, since cumulative detection probability asymptotes to 1.00. The most cost‐efficient effort for being able to reliably detect a 30% occupancy in a monitoring programme implemented across New South Wales with PARs is 147 sites with a deployment length of 18 days. This number of sites is feasible given the ease of deployment and the relative low cost of PAR units (Hill et al., 2018).

Camera trapping was moderately expensive because we deployed six cameras per site and because at least 100 sites were needed to have a high chance at detecting a change in occupancy. However, camera trapping could only detect at least 80% changes in koala occupancy with sufficient levels of power, even when the number of sites and deployment times was set at the maximum. Given the goal of biodiversity monitoring is often to detect small‐to‐moderate changes in target populations, camera trapping for the purpose of detecting changes in koala occupancy is relatively expensive and ineffective.

In contrast, drones have a large upfront equipment cost and can become expensive as the number of repeat surveys increases because staff are required in the field for each survey. However, these costs are offset by relatively low post‐processing costs given koala detections are made in real time. The cost per koala detection via drone in quadrat counts was found to be cheaper compared to spotlighting and spot‐assessment technique radial searches (Howell et al., 2021). This hints that drones may be a more cost‐effective method to obtain count‐based data in contrast to presence/absence data. Furthermore, technological advances may allow for more cost‐effective drone monitoring methods. For example, our cost estimates included on‐ground validations via field staff, including additional vehicle costs, but this may be unnecessary if drone‐based validation can occur by the pilot with a drone‐mounted spotlight.

### 4.4 Limitations and future directions

There were several limitations present within the study that would likely affect method‐specific detection performance and more broadly affect power to detect management‐relevant change in koala occupancy. Sampling methods did not occur concurrently at many sites. Sites located in the Pilliga (*n* = 10) experienced flooding conditions in the previous years which prevented PARs deployment in the preceding season, and hence, comparisons are made with these data from up to 3 years prior to drone and camera trap surveys. It is possible that koalas detected by PARs in the Pilliga could have immigrated or local extinction could have occurred, and hence, the koalas may not have been available for detection when drones and cameras were deployed. This is possible given declines in the Pilliga population have been previously noted (Lunney et al., 2017) and may be ongoing. The temporal mismatch is present across all sites, since PARs were generally used in the preceding season as it is necessary to deploy in spring/summer, yet drones require cold weather for optimum detection, which presents difficulties in direct comparisons of detection probability between methods due to immigration/emigration and colonisation/extinction processes.

Despite the clear advantages of PARs for monitoring koala occupancy, there are additional considerations when selecting a method for optimised surveys. While we have focused on detection probability as a single metric that can be used to evaluate survey method performance, some monitoring programmes or survey requirements may be time‐sensitive (e.g., pre‐harvest monitoring for risk reduction of koalas in logging operations), and hence passive devices that require post‐processing may be less ideal compared to drone surveys, which have instant data collection and do not require post‐processing. Continued development of semi‐automated artificial intelligence is expected to increase post‐processing efficiency (Ross et al., 2023; Wood et al., 2019). Additionally, the results we present are based on measuring the power to detect changes in occupancy and the associated costs; however, ecological monitoring programmes may require monitoring indicative of population abundance since it is a more sensitive metric to change. All three methods, with modifications to the sampling design, can potentially achieve abundance estimates. PARs can estimate abundance through a grid‐based arrangement where animal vocalisations can be incorporated into spatial count models (Law et al., 2021). Spatial count modelling can also be applied to camera trap data (Bengsen et al., 2022). Drones can estimate abundance through repeated quadrat counts which is then modelled with N‐mixture models (Brack et al., 2023). Any of the three methods may be suitable for abundance estimation using the model formulated by Royle and Nichols (2003). Further comparisons are required to determine which methods are the most effective for estimating koala abundance as a metric.

An important caveat for interpretation of the results is that the outcomes are dependent on the seasonal implementation of each method, which may not be optimal for a given method. Drone surveys and camera trap surveys were conducted in autumn and winter, and passive acoustic surveys were conducted during spring and early summer. The detection probability and power to detect a change in occupancy are likely to change if these survey methods are conducted in different seasons compared to what we have presented. Both drones and PARs were likely used in ideal conditions. Detection probability with drone surveys rapidly drops as the ambient air temperature increases, which is likely due to the environment becoming too warm for detections to be contrasted against their habitat (Kays et al., 2019). Since acoustic surveys rely on animal vocalisations and since koala vocalisations occur more frequently in spring and summer (Ellis et al., 2011), we expect detection probability to be low during autumn and winter using this method.

Lastly, this comparison was made between emerging methods, and other previously commonly used methods were not investigated. This includes spotlight transects, faecal pellet surveys and diurnal transects, which have all been previously used to detect koalas (Lunney et al., 1998; Martin, 1985; Wilmott et al., 2018). It is important that future studies test the power to detect decline and costs of implementation for these survey methods as well to as gain a completeness in identifying the most optimal method and monitoring design to detect change in koala populations. It is important that this approach is adopted to improve ecological monitoring of other threatened species.

## 5 CONCLUSION

We provide the first comparative assessment of thermal drones, PARs, and camera trapping at detecting changes in koala occupancy while accounting for survey costs. Our results suggest that PARs are the most cost‐effective method for detecting change in koala occupancy due to high detection probabilities and relatively low upfront and ongoing costs. Optimising the design of biodiversity surveys will help ensure that important changes in populations are detected with confidence and scarce conservation resources are spent most efficiently.

## AUTHOR CONTRIBUTIONS

**Chad T. Beranek:** Data curation (supporting); formal analysis (lead); investigation (supporting); visualization (lead); writing – original draft (lead); writing – review and editing (equal). **Darren Southwell:** Formal analysis (supporting); methodology (supporting); supervision (lead); validation (lead); writing – review and editing (equal). **Tim S. Jessop:** Data curation (lead); methodology (equal); project administration (equal); resources (equal); writing – review and editing (lead). **Benjamin Hope:** Investigation (lead); methodology (supporting); project administration (equal); resources (equal); writing – review and editing (supporting). **Veronica Fernandes Gama:** Data curation (lead); validation (equal). **Nicole Gallahar:** Investigation (lead); methodology (supporting); project administration (equal); resources (equal); writing – review and editing (equal). **Elliot Webb:** Investigation (supporting); project administration (supporting); resources (equal). **Brad Law:** Data curation (supporting); investigation (supporting); resources (supporting); writing – review and editing (equal). **Allen McIlwee:** Conceptualization (supporting); methodology (supporting); resources (supporting). **Jared Wood:** Data curation (equal); resources (supporting). **Adam Roff:** Conceptualization (supporting); data curation (supporting); methodology (supporting); project administration (supporting); writing – review and editing (supporting). **Graeme Gillespie:** Conceptualization (lead); methodology (lead); project administration (lead); resources (lead); writing – review and editing (equal).

## CONFLICT OF INTEREST STATEMENT

All authors declare no competing interests.

## Supporting information


Data S1:

## Data Availability

The data and analysis have been shared to Mendeley Data and can be accessed through the following link: https://doi.org/10.17632/87kkhjwfyg.1.

## References

[ece311659-bib-0001] Bengsen, A. J. , Forsyth, D. M. , Ramsey, D. S. , Amos, M. , Brennan, M. , Pople, A. R. , Comte, S. , & Crittle, T. (2022). Estimating deer density and abundance using spatial mark–resight models with camera trap data. Journal of Mammalogy, 103(3), 711–722. 10.1093/jmammal/gyac016 35707678 PMC9189690

[ece311659-bib-0002] Beranek, C. T. , Roff, A. , Denholm, B. , Howell, L. G. , & Witt, R. R. (2020). Trialling a real‐time drone detection and validation protocol for the koala (*Phascolarctos cinereus*). Australian Mammalogy, 43(2), 260–264. 10.1071/AM20043

[ece311659-bib-0003] Brack, I. V. , Kindel, A. , de Oliveira, L. F. B. , & Lahoz‐Monfort, J. J. (2023). Optimally designing drone‐based surveys for wildlife abundance estimation with N‐mixture models. Methods in Ecology and Evolution, 14(3), 898–910. 10.1111/2041-210X.14054

[ece311659-bib-0004] Catling, P. C. , Burt, R. J. , & Kooyman, R. (1997). A comparison of techniques used in a survey of the ground‐dwelling and arboreal mammals in forests in north‐eastern New South Wales. Wildlife Research, 24(4), 417–432. 10.1071/WR96073

[ece311659-bib-0005] Caughlan, L. , & Oakley, K. L. (2001). Cost considerations for long‐term ecological monitoring. Ecological Indicators, 1(2), 123–134. 10.1016/S1470-160X(01)00015-2

[ece311659-bib-0006] Cristescu, R. H. , Foley, E. , Markula, A. , Jackson, G. , Jones, D. , & Frere, C. (2015). Accuracy and efficiency of detection dogs: A powerful new tool for koala conservation and management. Scientific Reports, 5(1), 8349. 10.1038/srep08349 25666691 PMC4322364

[ece311659-bib-0008] di Stephano, J. (2003). How much power is enough? Against the development of an arbitrary convention for statistical power calculations. Functional Ecology, 17, 707–709. 10.1046/j.1365-2435.2003.00782.x

[ece311659-bib-0010] Ellis, W. , Bercovitch, F. , Fitzgibbon, S. , Roe, P. , Wimmer, J. , Melzer, A. , & Wilson, R. (2011). Koala bellows and their association with the spatial dynamics of free‐ranging koalas. Behavioral Ecology, 22(2), 372–377. 10.1093/beheco/arq216

[ece311659-bib-0011] Field, S. A. , Tyre, A. J. , & Possingham, H. P. (2005). Optimising allocation of monitoring effort under economic and observational constraints. The Journal of Wildlife Management, 69(2), 473–482. 10.2193/0022-541X(2005)069[0473:OAOMEU]2.0.CO;2

[ece311659-bib-0012] Fiske, I. , & Chandler, R. (2011). Unmarked: An R package for fitting hierarchical models of wildlife occurrence and abundance. Journal of Statistical Software, 43, 1–23. 10.18637/jss.v043.i10

[ece311659-bib-0013] Goldingay, R. L. , McHugh, D. , & Parkyn, J. L. (2022). Multiyear monitoring of threatened iconic arboreal mammals in a mid‐elevation conservation reserve in eastern Australia. Ecology and Evolution, 12(5), e8935. 10.1002/ece3.8935 35646314 PMC9130560

[ece311659-bib-0015] Guillera‐Arroita, G. , & Lahoz‐Monfort, J. J. (2012). Designing studies to detect differences in species occupancy: Power analysis under imperfect detection. Methods in Ecology and Evolution, 3(5), 860–869. 10.1111/j.2041-210X.2012.00225.x

[ece311659-bib-0016] Hagens, S. V. , Rendall, A. R. , & Whisson, D. A. (2018). Passive acoustic surveys for predicting species' distributions: Optimising detection probability. PLoS One, 13(7), e0199396. 10.1371/journal.pone.0199396 30020938 PMC6051584

[ece311659-bib-0017] Han, Y. G. , Cho, Y. , & Kwon, O. (2015). The use of conservation drones in ecology and wildlife research. Journal of Ecology and Environment, 38(1), 113–118. 10.5141/ecoenv.2015.012

[ece311659-bib-0018] Heiniger, J. , Cameron, S. F. , Madsen, T. , Niehaus, A. C. , & Wilson, R. S. (2020). Demography and spatial requirements of the endangered northern quoll on Groote Eylandt. Wildlife Research, 47(3), 224–238. 10.1071/WR19052

[ece311659-bib-0019] Hill, A. P. , Prince, P. , Piña Covarrubias, E. , Doncaster, C. P. , Snaddon, J. L. , & Rogers, A. (2018). AudioMoth: Evaluation of a smart open acoustic device for monitoring biodiversity and the environment. Methods in Ecology and Evolution, 9(5), 1199–1211. 10.1111/2041-210X.12955

[ece311659-bib-0020] Howell, L. G. , Clulow, J. , Jordan, N. R. , Beranek, C. T. , Ryan, S. A. , Roff, A. , & Witt, R. R. (2021). Drone thermal imaging technology provides a cost‐effective tool for landscape‐scale monitoring of a cryptic forest‐dwelling species across all population densities. Wildlife Research, 49(1), 66–78. 10.1071/WR21034

[ece311659-bib-0021] Kays, R. , Sheppard, J. , Mclean, K. , Welch, C. , Paunescu, C. , Wang, V. , Kravit, G. , & Crofoot, M. (2019). Hot monkey, cold reality: Surveying rainforest canopy mammals using drone‐mounted thermal infrared sensors. International Journal of Remote Sensing, 40(2), 407–419. 10.1080/01431161.2018.1523580

[ece311659-bib-0022] Kellner, K. F. , & Swihart, R. K. (2014). Accounting for imperfect detection in ecology: A quantitative review. PLoS One, 9(10), e111436. 10.1371/journal.pone.0111436 25356904 PMC4214722

[ece311659-bib-0023] Kéry, M. (2002). Inferring the absence of a species: A case study of snakes. The Journal of Wildlife Management, 66(2), 330–338. 10.2307/3803165

[ece311659-bib-0024] Lahoz‐Monfort, J. J. , & Magrath, M. J. (2021). A comprehensive overview of technologies for species and habitat monitoring and conservation. Bioscience, 71(10), 1038–1062. 10.1093/biosci/biab073 34616236 PMC8490933

[ece311659-bib-0025] Law, B. , Gonsalves, L. , Bilney, R. , Peterie, J. , Pietsch, R. , Roe, P. , & Truskinger, A. (2020). Using passive acoustic recording and automated call identification to survey koalas in the southern forests of New South Wales. Australian Zoologist, 40, 477–486. 10.7882/AZ.2019.033

[ece311659-bib-0027] Law, B. , Gonsalves, L. , Kerr, I. , McConville, A. , & Tap, P. (2021). Koala Monitoring in State Forests. Report prepared for DPIE. NSW Department of Primary Industries (Forest Science).

[ece311659-bib-0028] Law, B. S. , Brassil, T. , Gonsalves, L. , Roe, P. , Truskinger, A. , & McConville, A. (2018). Passive acoustics and sound recognition provide new insights on status and resilience of an iconic endangered marsupial (koala *Phascolarctos cinereus*) to timber harvesting. PLoS One, 13(10), e0205075. 10.1371/journal.pone.0205075 30379836 PMC6209150

[ece311659-bib-0029] Law, B. S. , Gonsalves, L. , Burgar, J. , Brassil, T. , Kerr, I. , & O'Loughlin, C. (2022a). Fire severity and its local extent are key to assessing impacts of Australian mega‐fires on koala (*Phascolarctos cinereus*) density. Global Ecology and Biogeography, 31(4), 714–726. 10.1111/geb.13458

[ece311659-bib-0030] Lindenmayer, D. B. , Gibbons, P. , Bourke, M. A. X. , Burgman, M. , Dickman, C. R. , Ferrier, S. , Fitzsimons, J. , Freudenberger, D. , Garnett, S. T. , Groves, C. , & Hobbs, R. J. (2012). Improving biodiversity monitoring. Austral Ecology, 37(3), 285–294. 10.1111/j.1442-9993.2011.02314.x

[ece311659-bib-0031] Lunney, D. , Phillips, S. , Callaghan, J. , & Coburn, D. (1998). Determining the distribution of koala habitat across a shire as a basis for conservation: A case study from Port Stephens, New South Wales. Pacific Conservation Biology, 4(3), 186–196. 10.1071/PC980186

[ece311659-bib-0032] Lunney, D. , Predavec, M. , Sonawane, I. , Kavanagh, R. , Barrott‐Brown, G. , Phillips, S. , Callaghan, J. , Mitchell, D. , Parnaby, H. , Paull, D. C. , & Shannon, I. (2017). The remaining koalas (*Phascolarctos cinereus*) of the Pilliga forests, north‐west New South Wales: Refugial persistence or a population on the road to extinction? Pacific Conservation Biology, 23(3), 277–294. 10.1071/PC17008

[ece311659-bib-0033] Lunney, D. , Stalenberg, E. , Santika, T. , & Rhodes, J. R. (2014). Extinction in Eden: Identifying the role of climate change in the decline of the koala in south‐eastern NSW. Wildlife Research, 41(1), 22–34. 10.1071/WR13054

[ece311659-bib-0034] MacKenzie, D. I. , Nichols, J. D. , Lachman, G. B. , Droege, S. , Royle, A. J. , & Langtimm, C. A. (2002). Estimating site occupancy rates when detection probabilities are less than one. Ecology, 83, 2248–2255. 10.1890/0012-9658(2002)083[2248:ESORWD]2.0.CO;2

[ece311659-bib-0036] MacKenzie, D. I. , & Royle, J. A. (2005). Designing occupancy studies: General advice and allocating survey effort. Journal of Applied Ecology, 42(6), 1105–1114. 10.1111/j.1365-2664.2005.01098.x

[ece311659-bib-0037] Martin, R. W. (1985). Overbrowsing, and decline of a population of the koala, *Phascolarctos cinereus*, in Victoria. I. Food preference and food tree defoliation. Wildlife Research, 12(3), 355–365. 10.1071/WR9850355

[ece311659-bib-0038] McAlpine, C. , Lunney, D. , Melzer, A. , Menkhorst, P. , Phillips, S. , Phalen, D. , Ellis, W. , Foley, W. , Baxter, G. , De Villers, D. , Kavanagh, R. , Adams‐Hosking, C. , Todd, C. , Whisson, D. , Molsher, R. , Walter, M. , Lawler, I. , & Close, R. (2015). Conserving koalas in the 21st century: Regional trends, challenges and prognoses. Biological Conservation, 192, 226–236. 10.1016/j.biocon.2015.09.020

[ece311659-bib-0039] Mills, D. , Fattebert, J. , Hunter, L. , & Slotow, R. (2019). Maximising camera trap data: Using attractants to improve detection of elusive species in multi‐species surveys. PLoS One, 14(5), e0216447. 10.1371/journal.pone.0216447 31141506 PMC6541258

[ece311659-bib-0040] Moore, H. A. , Dunlop, J. A. , Geyle, H. M. , Greenwood, L. , & Nimmo, D. G. (2023). First you get the money, then you get the power: Comparing the cost and power of monitoring programs to detect changes in occupancy of a threatened marsupial predator. Conservation Science and Practice, 5(2), e12881. 10.1111/csp2.12881

[ece311659-bib-0041] Munks, S. A. , Corkrey, R. , & Foley, W. J. (1996). Characteristics of arboreal marsupial habitat in the semi‐arid woodlands of northern Queensland. Wildlife Research, 23(2), 185–195. 10.1071/WR9960185

[ece311659-bib-0042] Ross, S.‐J. , O'Connell, D. P. , Deichmann, J. L. , Desjonquères, C. , Gasc, A. , Phillips, J. N. , Sethi, S. S. , Wood, C. M. , & Burivalova, Z. (2023). Passive acoustic monitoring provides a fresh perspective on fundamental ecological questions. Functional Ecology, 37, 959–975. 10.1111/1365-2435.14275

[ece311659-bib-0043] Royle, J. A. , & Nichols, J. D. (2003). Estimating abundance from repeated presence–absence data or point counts. Ecology, 84(3), 777–790. 10.1890/0012-9658(2003)084[0777:EAFRPA]2.0.CO;2

[ece311659-bib-0044] Ryan, M. A. , Whisson, D. A. , Holland, G. J. , & Arnould, J. P. (2013). Activity patterns of free‐ranging koalas (*Phascolarctos cinereus*) revealed by accelerometry. PLoS One, 8(11), e80366. 10.1371/journal.pone.0080366 24224050 PMC3817117

[ece311659-bib-0047] Shumway, N. , Lunney, D. , Seabrook, L. , & McAlpine, C. (2015). Saving our national icon: An ecological analysis of the 2011 Australian senate inquiry into status of the koala. Environmental Science & Policy, 54, 297–303. 10.1016/j.envsci.2015.07.024

[ece311659-bib-0048] Stephenson, P. J. (2020). Technological advances in biodiversity monitoring: Applicability, opportunities and challenges. Current Opinion in Environmental Sustainability, 45, 36–41. 10.1016/j.cosust.2020.08.005

[ece311659-bib-0049] Stokeld, D. , Frank, A. S. , Hill, B. , Choy, J. L. , Mahney, T. , Stevens, A. , Young, S. , Rangers, D. , Rangers, W. , & Gillespie, G. R. (2015). Multiple cameras required to reliably detect feral cats in northern Australian tropical savanna: An evaluation of sampling design when using camera traps. Wildlife Research, 42(8), 642–649. 10.1071/WR15083

[ece311659-bib-0051] Tuia, D. , Kellenberger, B. , Beery, S. , Costelloe, B. R. , Zuffi, S. , Risse, B. , Mathis, A. , Mathis, M. W. , van Langevelde, F. , Burghardt, T. , & Kays, R. (2022). Perspectives in machine learning for wildlife conservation. Nature Communications, 13(1), 792. 10.1038/s41467-022-27980-y PMC8828720 35140206

[ece311659-bib-0052] Whisson, D. A. , & Ashman, K. R. (2020). When an iconic native animal is overabundant: The koala in southern Australia. Conservation Science and Practice, 2(5), 188. 10.1111/csp2.188

[ece311659-bib-0053] Whisson, D. A. , Dixon, V. , Taylor, M. L. , & Melzer, A. (2016). Failure to respond to food resource decline has catastrophic consequences for koalas in a high‐density population in southern Australia. PLoS One, 11(1), e0144348. 10.1371/journal.pone.0144348 26735846 PMC4703219

[ece311659-bib-0054] Wilmott, L. , Cullen, D. , Madani, G. , Krogh, M. , & Madden, K. (2018). Are koalas detected more effectively by systematic spotlighting or diurnal searches? Australian Mammalogy, 41(1), 157–160. 10.1071/AM18006

[ece311659-bib-0055] Witt, R. R. , Beranek, C. T. , Howell, L. G. , Ryan, S. A. , Clulow, J. , Jordan, N. R. , Denholm, B. , & Roff, A. (2020). Real‐time drone derived thermal imagery outperforms traditional survey methods for an arboreal forest mammal. PLoS One, 15(11), e0242204. 10.1371/journal.pone.0242204 33196649 PMC7668579

[ece311659-bib-0056] Wood, C. M. , Popescu, V. D. , Klinck, H. , Keane, J. J. , Gutiérrez, R. J. , Sawyer, S. C. , & Peery, M. Z. (2019). Detecting small changes in populations at landscape scales: A bioacoustic site‐occupancy framework. Ecological Indicators, 98, 492–507. 10.1016/j.ecolind.2018.11.018

