# Supplementary material for: Comparing the cost‐effectiveness of drones, camera trapping and passive acoustic recorders in detecting changes in koala occupancy
Source: Ecol Evol. 2024 Jul 2;14(7):e11659. doi: 10.1002/ece3.11659 (PMC11219196; doi:10.1002/ece3.11659)
Supplement: Supplementary file 1 — Data S1: [file ECE3-14-e11659-s001.docx]

**Supplementary material 1.**

**Table S1.** Summary of acoustic monitoring sites selected.

| **Study area** | **Organisation** **(contact)** | **No. sites available** | **No. sites surveyed** | **Pres/Abs data** | **Recent years of survey/Notes** |
| --- | --- | --- | --- | --- | --- |
| North-east Coast^1^ | DPI Forest Science  (B. Law) | 2020: 69 2021: 81 | 14 | yes no | 2020 2021 |
| Richmond Range^1^ | Southern Cross University  (R. Goldingay) | 2021: 36 | 4 | yes | 2021 |
| Pilliga State Forests | Forestry Corporation NSW (P. Tap) | 2018: 96  2019: 96  2020: 94 | 10 | yes | 2018–2019 (2020 no koalas detected,  2021 no survey, flooded,  2022 no survey, flooded) |
| Southern Highlands | DPE  (L. Wilmott) | 2021: 56 | 2 | yes | 2021–two acoustic arrays |
| Kosciusko NP | ANU/NPWS (K. Marsh) | 2021-22: 64 | 8 | yes | 2021–22 |
| **TOTAL** |  |  | **38** |  |  |

^1^North-east Coast and Richmond Range are considered as one study area due to proximity.

**Table S2.** Summary of all models tested. Temp = Maximum air temperature (°C). rh = relative humidity (%). rain = daily total rainfall (mm). Method = method used, either acoustics, camera, or drone. *β*_0_ = probability of detection intercept. *α*_0​_ = probability of occupancy intercept.

| Model abbreviation | Model name | Model intercepts |
| --- | --- | --- |
| M1: Null model – p_(.)_ ψ_(.)_ | Null model | logit(*p_ij_*​) = *β*_0_​  logit(*ψ_i_*_​_) = *α*_0​_ |
| M2: p(_method_) ψ_(.)_ | Detection per method | logit(*p_ij_*​) = *β*_0_​ + *β*_1_​⋅Method(Camera)*_j_*​ + *β*_2_​ ⋅Method(Drone)*_j_*​  logit(*ψ_i_*_​_) = *α*_0​_ |
| M3: p(_method*rain_) ψ_(.)_ | Interaction between method and linear rain | logit(*p_ij_*​) = *β*_0_​ + *β*_1_​⋅Method(Camera)*_j_*​ + *β*_2_​ ⋅Method(Drone)*_j_*​ + *β*_3_​⋅rain*_i_*_​_ + *β*_4_​⋅Method(Camera)*_j_*​⋅rain*_i_*_​_ + *β*_5_⋅Method(Drone)*_j_*​⋅rain*_i_*_​_  logit(*ψ_i_*_​_) = *α*_0​_ |
| M4: p(_method*rh_) ψ_(.)_ | Interaction between method and linear rh | logit(*p_ij_*​) = *β*_0_​ + *β*_1_​⋅Method(Camera)*_j_*​ + *β*_2_​ ⋅Method(Drone)*_j_*​ + *β*_3_​⋅rh*_i_*_​_ + *β*_4_​⋅Method(Camera)*_j_*​⋅rh*_i_*_​_ + *β*_5_⋅Method(Drone)*_j_*​⋅rh*_i_*_​_  logit(*ψ_i_*_​_) = *α*_0​_ |
| M5: p(_method*rh2_) ψ_(.)_ | Interaction between method and quadratic rh | logit(*p_ij_*​)=*β*_0_​ + *β*_1_​⋅Method(Camera)*_j_*_​_ + *β*_2_​⋅Method(Drone)*_j_*_​_ + *β*_3_​⋅(rh*_i_*)^2^ + *β*_4_​⋅Method(Camera)*_j_*​⋅(rh*_i_*​)^2^ + *β*_5_​⋅Method(Drone)*_j_*_​_⋅(rh*_i_*)^2^  logit(*ψ_i_*_​_) = *α*_0​_ |
| M6: p(_method*temp_) ψ_(.)_ | Interaction between method and linear temp | logit(*p_ij_*​) = *β*_0_​ + *β*_1_​⋅Method(Camera)*_j_*​ + *β*_2_​ ⋅Method(Drone)*_j_*​ + *β*_3_​⋅temp*_i_*_​_ + *β*_4_​⋅Method(Camera)*_j_*​⋅temp*_i_*_​_ + *β*_5_⋅Method(Drone)*_j_*​⋅temp*_i_*_​_  logit(*ψ_i_*_​_) = *α*_0​_ |
| M7: p(_method*temp2_) ψ_(.)_ | Interaction between method and quadratic temp | logit(*p_ij_*​)=*β*_0_​ + *β*_1_​⋅Method(Camera)*_j_*_​_ + *β*_2_​⋅Method(Drone)*_j_*_​_ + *β*_3_​⋅(temp*_i_*)^2^ + *β*_4_​⋅Method(Camera)*_j_*​⋅(temp*_i_*​)^2^ + *β*_5_​⋅Method(Drone)*_j_*_​_⋅(temp*_i_*)^2^  logit(*ψ_i_*_​_) = *α*_0​_ |
| M8: p(_method*rain+method*rh_) ψ_(.)_ | Additive effect of two interactions: interaction with method and linear rain, an interaction between method and linear rh | logit(*p_ij_*​) = *β*_0_​ + *β*_1_​⋅Method(Camera)*_j_*​ + *β*_2_​ ⋅Method(Drone)*_j_*​ + *β*_3_​⋅rain*_i_*_​_ + *β*_4_​⋅Method(Camera)*_j_*​⋅rain*_i_*_​_ + *β*_5_⋅Method(Drone)*_j_*​⋅rain*_i_* + *β*_6_​⋅rh*_i_*_​_ + *β*_7_​⋅Method(Camera)*_j_*​⋅rh*_i_*_​_ + *β*_8_⋅Method(Drone)*_j_*​⋅rh*_i_*_​_  logit(*ψ_i_*_​_) = *α*_0​_ |
| M9: p(_method*rain+method*rh2_) ψ_(.)_ | Additive effect of two interactions: interaction with method and linear rain, an interaction between method and quadratic rh | logit(*p_ij_*​) = *β*_0_​ + *β*_1_​⋅Method(Camera)*_j_*​ + *β*_2_​ ⋅Method(Drone)*_j_*​ + *β*_3_​⋅rain*_i_*_​_ + *β*_4_​⋅Method(Camera)*_j_*​⋅rain*_i_*_​_ + *β*_5_⋅Method(Drone)*_j_*​⋅rain*_i_* + *β*_6_​⋅(rh*_i_*_​_)^2^ + *β*_7_​⋅Method(Camera)*_j_*​⋅(rh*_i_*​)^2^ + *β*_8_​⋅Method(Drone)*_j_*​⋅(rh*_i_*​)^2^  logit(*ψ_i_*_​_) = *α*_0​_ |
| M10: p(_method*rain+method*temp_) ψ_(.)_ | Additive effect of two interactions: interaction with method and linear rain, an interaction between method and linear temp | logit(*p_ij_*​) = *β*_0_​ + *β*_1_​⋅Method(Camera)*_j_*​ + *β*_2_​ ⋅Method(Drone)*_j_*​ + *β*_3_​⋅rain*_i_*_​_ + *β*_4_​⋅Method(Camera)*_j_*​⋅rain*_i_*_​_ + *β*_5_⋅Method(Drone)*_j_*​⋅rain*_i_* + *β*_6_⋅temp*_i_*_​_ + *β*_7_​⋅Method(Camera)*_j_*​⋅temp*_i_*_​_ + *β*_8_⋅Method(Drone)*_j_*​⋅temp*_i_*_​_  logit(*ψ_i_*_​_) = *α*_0​_ |
| M11: p(_method*rain+method*temp2_) ψ_(.)_ | Additive effect of two interactions: interaction with method and linear rain, an interaction between method and quadratic temp | logit(*p_ij_*​) = *β*_0_​ + *β*_1_​⋅Method(Camera)*_j_*​ + *β*_2_​ ⋅Method(Drone)*_j_*​ + *β*_3_​⋅rain*_i_*_​_ + *β*_4_​⋅Method(Camera)*_j_*​⋅rain*_i_*_​_ + *β*_5_⋅Method(Drone)*_j_*​⋅rain*_i_* + *β*_6_​⋅(temp*_i_*)^2^ + *β*_7_​⋅Method(Camera)*_j_*​⋅(temp*_i_*​)^2^ + *β*_8_​⋅Method(Drone)*_j_*_​_⋅(temp*_i_*)^2^  logit(*ψ_i_*_​_) = *α*_0​_ |
| M12: p(_method*rh+method*temp_) ψ_(.)_ | Additive effect of two interactions: interaction with method and linear rh, an interaction between method and linear temp | logit(*p_ij_*​) = *β*_0_​ + *β*_1_​⋅Method(Camera)*_j_*​ + *β*_2_​ ⋅Method(Drone)*_j_*​ + *β*_3_​⋅rh*_i_*_​_ + *β*_4_​⋅Method(Camera)*_j_*​⋅rh*_i_*_​_ + *β*_5_⋅Method(Drone)*_j_*​⋅rh*_i_* + *β*_6_​⋅temp*_i_*_​_ + *β*_7_⋅Method(Camera)*_j_*​⋅temp*_i_*_​_ + *β*_8_⋅Method(Drone)*_j_*​⋅temp*_i_*_​_  logit(*ψ_i_*_​_) = *α*_0​_ |
| M13: p(_method*rh+method*temp2_) ψ_(.)_ | Additive effect of two interactions: interaction with method and linear rh, an interaction between method and quadratic temp | logit(*p_ij_*​) = *β*_0_​ + *β*_1_​⋅Method(Camera)*_j_*​ + *β*_2_​ ⋅Method(Drone)*_j_*​ + *β*_3_​⋅rh*_i_*_​_ + *β*_4_​⋅Method(Camera)*_j_*​⋅rh*_i_*_​_ + *β*_5_⋅Method(Drone)*_j_*​⋅rh*_i_*_​_ + *β*_6_​⋅(temp*_i_*)^2^ + *β*_7_​⋅Method(Camera)*_j_*​⋅(temp*_i_*​)^2^ + *β*_8_​⋅Method(Drone)*_j_*_​_⋅(temp*_i_*)^2^  logit(*ψ_i_*_​_) = *α*_0​_ |
| M14: p(_method*rh2+method*temp_) ψ_(.)_ | Additive effect of two interactions: interaction with method and quadratic rh, an interaction between method and linear temp | logit(*p_ij_*​)=*β*_0_​ + *β*_1_​⋅Method(Camera)*_j_*_​_ + *β*_2_​⋅Method(Drone)*_j_*_​_ + *β*_3_​⋅(rh*_i_*)^2^ + *β*_4_​⋅Method(Camera)*_j_*​⋅(rh*_i_*​)^2^ + *β*_5_​⋅Method(Drone)*_j_*_​_⋅(rh*_i_*)^2^ + *β*_6_​⋅temp*_i_*_​_ + *β*_7_⋅Method(Camera)*_j_*​⋅temp*_i_*_​_ + *β*_8_⋅Method(Drone)*_j_*​⋅temp*_i_*_​_  logit(*ψ_i_*_​_) = *α*_0​_ |
| M15: p(_method*rh2+method*temp2_) ψ_(.)_ | Additive effect of two interactions: interaction with method and quadratic rh, an interaction between method and quadratic temp | logit(*p_ij_*​)=*β*_0_​ + *β*_1_​⋅Method(Camera)*_j_*_​_ + *β*_2_​⋅Method(Drone)*_j_*_​_ + *β*_3_​⋅(rh*_i_*)^2^ + *β*_4_​⋅Method(Camera)*_j_*​⋅(rh*_i_*​)^2^ + *β*_5_​⋅Method(Drone)*_j_*_​_⋅(rh*_i_*)^2^ + *β*_6_​⋅(temp*_i_*)^2^ + *β*_7_​⋅Method(Camera)*_j_*​⋅(temp*_i_*​)^2^ + *β*_8_​⋅Method(Drone)*_j_*_​_⋅(temp*_i_*)^2^  logit(*ψ_i_*_​_) = *α*_0​_ |

**Table S3.** Summary of costs. Assuming a standard cost of $100/hr for personnel time. Since AudioMoth and Song Meters (SM4) were used interchangeably, an average cost of these devices was used to determine the cost of acoustic equipment ([$899+$218]/2 = $558.50). We assume camera trap deployment is one site setup per 3 hours and one site can be collected in 2 hours (including mean travel distances between sites). We also assume both deployment and collection for acoustics is 2 hrs each. We assume that drones take 2-hrs to survey a 25-ha site, and that four surveys can be completed in one night. Accommodation, food and travel costs are divided by the length of time it takes to do one site divided by 8 hrs (assuming an 8-hr workday). Accommodation is assumed to be $50.00 per person/8-hr workday, food is assumed to be $50.00 per person/8-hr workday and vehicles are assumed to be $400/8-hr workday. Drone surveys require two vehicles, one for the validation team and one for the drone team. UC = Upfront costs. PS = per site costs. PSS = per site per survey costs.

| Costs | Type | Camera | Drone | PAR |
| --- | --- | --- | --- | --- |
| Base costs (equipment startup) | Upfront | **$0** | **$21,732.00**  (Two DJI MEA2 [$9403 each], 14 M2E batteries [$209 each]) | **$0** |
| Training – Person hrs | Upfront | **$4,800.00**  ($100*16hrs*3people [two trainees and one trainer]) | **$12,000.00**  ($100*40hrs*3people [two trainees and one trainer]) | **$4,800.00**  ($100*16hrs*3people [two trainees and one trainer]) |
| Training – Accreditation | Upfront | **$0** | **$4,796.00**  (Two Remotely piloted aircraft [$1999] aeronautical radio operators’ certification [$399]) | **$0** |
| Total (UC) | Upfront | **$4,800.00** | **$38,528.00** | **$4,800.00** |
| Equipment per site | Per site | **$2,982.00**  (Six Reconyx HP2W Hyperfire 2 [$460 each), six SD cards [$25 each], 72 batteries [$1 each]) | **$50.00**  (Two SD cards [$25 each) | **$591.00**  (One acoustic device [$558 each], one SD card [$25 each], 4 batteries [$2 each]) |
| Planning | Per site | **$300.00**  ($100*3hrs) | **$400.00**  ($100*4hrs) | **$300.00**  ($100*3hrs) |
| Personnel costs | Per site | **$1,000.00**  ($100*3hrs deploy*2people + $100*2hrs collect*2people) | **$0 (this cost is per survey)** | **$800.00**  ($100*2hrs deploy*2people + $100*2hrs collect*2people) |
| Maintenance | Per site | **$200.00**  ($100*2hrs) | **$50.00**  ($100*0.5hrs) | **$50.00**  ($100*0.5hrs) |
| Travel costs (vehicle/food/ accommodation) | Per site | **$375.00**  (Deployment: [Accommodation: $50*2people/2.67 + food: $50*2people/2.67 + vehicle: $400/2.67] + collection [Accommodation: $50*2people/4 + food: $50*2people/4+ vehicle: $400/4]) | **$0 (this cost is per survey)** | **$300.00**  ([Accommodation: $50*2people/4 + food: $50*2people/4 + vehicle: $400/4] * 2 to cover deployment and collection) |
| Total (CS) | Per site | **$4,857.00** | **$500.00** | **$2,041.00** |
| Post processing | Per survey | **$4.17**  ($100/hr*2.5mins*1person) | **$0** | **$8.33**  ($100hr*5mins*1person) |
| Personnel costs | Per survey |  | **$,800.00**  ($100*2hrs*2 drone operators + $100*2hrs*2 validators) |  |
| Travel costs (vehicle/food/ accommodation) | Per survey | **$0 (this cost per site)** | **$300.00**  (Accommodation: $50*4people/4 + food: $50*4people/4 + two vehicles: $400/4*2) | **$0 (this cost is per site)** |
| Total (CSS) | Per survey | **$4.17** | **$1,100.00** | **$8.33** |

Supplementary material 2.

**TableS4.** Goodness of fit tests for the top performing models and the null model. Number of bootstrap simulations = 10,000. Bolding indicates tests that did not pass the 0.05 confidence limit.

| Model | Test | t0 | Mean | SD | P-value |
| --- | --- | --- | --- | --- | --- |
| p_(method*temp2)_ ψ_(.)_ | SSE | 125 | -4.33 | 18.9 | 0.59 |
|  | Freeman Tukey | 201 | -0.81 | 25.7 | 0.52 |
| Null - p_(.)_ ψ_(.)_ | SSE | 137 | 9.81 | 20.9 | 0.31 |
|  | Freeman-Tukey | 227 | 7.40 | 32.7 | 0.41 |


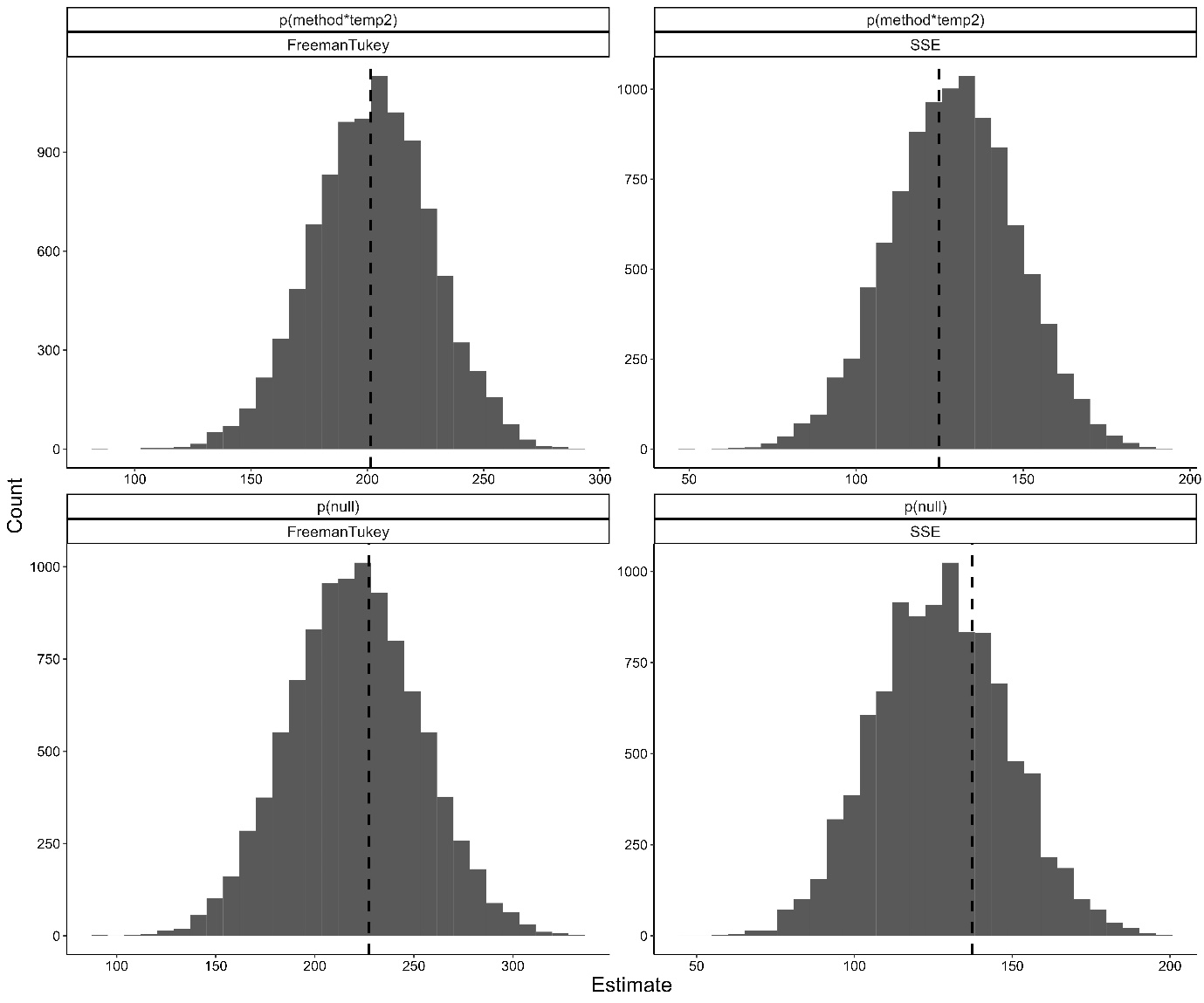


**Fig. S1.** Comparison of bootstrap histograms to observed means among and between models and tests. Number of bootstrap simulations = 10,000 per test. Dashed line indicates observed mean. Red dashed line indicates where the observed mean is outside the 95% distribution of the predicted values, indicating a lack of fit.

Supplementary material 3.

**TableS5.** Ranking all 22 models evaluating the effects of different covariates on koala detection probability estimated using single-season occupancy models. Column headings include n, the number of estimated parameters. AICc, Akaike Information Criterion; ΔAICc, difference between the AICc of the target model and the most parsimonious model. See Table S1S2 for model descriptions.

| Model | n | AICc | ΔAICc | Cumulative weight | Weight |
| --- | --- | --- | --- | --- | --- |
| M7: p(_method*temp2_) ψ_(.)_ | 7 | 926.06 | 0 | 0.61 | 0.61 |
| M6: p(_method*temp_) ψ_(.)_ | 7 | 928.47 | 2.41 | 0.79 | 0.18 |
| M4: p(_method*rh_) ψ_(.)_ | 7 | 930.25 | 4.19 | 0.87 | 0.08 |
| M2: p(_method_) ψ_(.)_ | 4 | 930.91 | 4.85 | 0.92 | 0.05 |
| M11: p(_method*rain+method*temp2_) ψ_(.)_ | 10 | 932.28 | 6.22 | 0.95 | 0.03 |
| M12: p(_method*rh+method*temp_) ψ_(.)_ | 10 | 933.68 | 7.62 | 0.96 | 0.01 |
| M15: p(_method*rh2+method*temp2_) ψ_(.)_ | 10 | 934.28 | 8.22 | 0.97 | 0.01 |
| M13: p(_method*rh+method*temp2_) ψ_(.)_ | 10 | 934.62 | 8.56 | 0.98 | 0.01 |
| M14: p(_method*rh2+method*temp_) ψ_(.)_ | 10 | 935.38 | 9.32 | 0.99 | 0.01 |
| M10: p(_method*rain+method*temp_) ψ_(.)_ | 10 | 935.68 | 9.62 | 0.99 | 0 |
| M8: p(_method*rain+method*rh_) ψ_(.)_ | 10 | 936.45 | 10.39 | 0.99 | 0 |
| M5: p(_method*rh2_) ψ_(.)_ | 7 | 936.79 | 10.73 | 1.00 | 0 |
| M3: p(_method*rain_) ψ_(.)_ | 7 | 937.02 | 10.96 | 1.00 | 0 |
| M9: p(_method*rain+method*rh2_) ψ_(.)_ | 10 | 944.7 | 18.64 | 1.00 | 0 |
| M1: Null model – p_(.)_ ψ_(.)_ | 2 | 1202.45 | 279.39 | 1.00 | 0 |

Graphical abstract

This study evaluates the cost-effectiveness of three sampling methods (thermal drones, passive acoustic recorders, and camera trapping) for detecting changes in koala occupancy. Analyzing data from 46 sites over 2018-2022, the study found that passive acoustic recorders deployed for 14 days at 148 sites provided the most cost-efficient method (80% power to detect a 30% decline in occupancy). The study recommends passive acoustic recorders as the preferred sampling method for monitoring koala occupancy compared to drones or cameras.
